# Investigation of the minimum uptake time for quantitative jawbone SPECT

**DOI:** 10.1186/s12903-025-07029-4

**Published:** 2025-10-14

**Authors:** Hironobu Hata, Kenji Hirata, Satoshi Shimomura, Kenji Imamachi, Takuya Asaka, Mayumi Kamaguchi, Kenta Miwa, Masashi Matsuzaka, Yoichi Mori, Toshikazu Nambu

**Affiliations:** 1 Department of Dentistry and Oral Surgery,, NHO Hokkaido Cancer Center, Sapporo, Japan; 2 Department of Diagnostic Imaging, Graduate School of Medicine, Hokkaido University, Sapporo, Japan; 3 Department of Radiology, NHO Hokkaido Cancer Center, Sapporo, Japan; 4 Department of Oral Pathobiological Science, Oral Diagnosis and Medicine, Faculty of Dental Medicine, Hokkaido University, Sapporo, Japan; 5 Department of Radiological Sciences, School of Health Sciences, Fukushima Medical University, Fukushima, Japan; 6 Clinical Research Support Centre, Hirosaki University Hospital, Hirosaki, Japan; 7 Department of Medical Informatics, Hirosaki University Hospital, Hirosaki, Japan

**Keywords:** SPECT, Quantitative analysis, MRONJ, Uptake time, Jawbone

## Abstract

**Background:**

The range of imaging times recommended for bone scintigraphy is wide, and uptake times vary across facilities. In our previous quantitative evaluation of jawbone SPECT using [^99m^Tc] Tc-HMDP, we reported that standardized uptake values at the 2-hour uptake time were comparable to those at the 3-hour uptake time. A reduction in the uptake time is of particular benefit to patients with poor general health conditions, such as those suffering from cancer. This study aimed to conduct additional research based on the hypothesis that further reduction of uptake time may be possible.

**Methods:**

Forty patients scheduled for jawbone SPECT at our hospital’s Department of Dentistry and Oral Surgery were enrolled in this study and assigned to two groups. The 1.0-hour uptake time group was imaged at two time points, 1.0 and 2.0 h, and the 1.5-hour uptake time group was imaged at two time points, 1.5 and 2.0 h, for jawbone lesions and parietal bone. We investigated statistically significant differences by comparing the maximum standardized uptake values (SUVmax) for each of the two groups using quantitative SPECT software.

**Results:**

When the SUVmax of the 1.0-hour uptake time group was compared, the median SUVmax of the parietal bone was not significantly different. In contrast, the median SUVmax of jawbone lesions significantly increased to 6.40 and 8.10, respectively (*p* < 0.001). Similarly, in the 1.5-hour uptake time group, the median SUVmax of the parietal bone was not significantly different. The median SUVmax of the jawbone lesions increased to 7.22 and 8.03, respectively, with a statistically significant difference (*p* < 0.001).

**Conclusions:**

The uptake time required for quantitative SPECT evaluation of jawbone lesions using [^99m^Tc] Tc-HMDP could not be further reduced from 2 h in 30-minute increments, and the shortest acceptable acquisition time for quantitative jawbone SPECT was 2 h.

## Background

Bone scintigraphy and quantitative single-photon emission computed tomography (SPECT) have recently been used to assess inflammatory jaw lesions, including osteoradionecrosis, osteomyelitis, and medication-related osteonecrosis of the jaw (MRONJ) [1–8]. Current bone scintigraphy guidelines provide a wide range of permissible uptake durations for delayed-phase imaging (i.e., 2–4 h after the injection of technetium-99 m radiopharmaceuticals) [9, 10]. Therefore, the imaging time (i.e., uptake time) varied across facilities. In the quantitative analysis of the bone scintigraphy data, it has not been confirmed whether the SUV is stable within the wide uptake time tolerance range indicated by these guidelines. In a previous study, we reported that the SUVmax at a 2-hour uptake time was equivalent to that at the 3-hour uptake time, with no clinical difference according to [^99m^Tc] Tc-HMDP quantitative SPECT of the jawbone [11]. A reduction of as much as one hour in the waiting time before imaging can greatly reduce the burden on patients who may be in poor physical condition. The medical interview form for Clearbone ([^99m^Tc] Tc-HMDP; Nihon Medi-Physics) [12] preparation states that a 1-hour uptake time is possible, but it is not certain that a stable quantitative evaluation of SPECT is possible with a 1-hour uptake time. We performed additional investigations to test the hypothesis that the uptake time for [^99m^Tc] Tc-HMDP quantitative SPECT of the jawbone can be further reduced from 2 h.

## Methods

### Patients and study design

Forty patients who underwent SPECT imaging of the jaw and were diagnosed with MRONJ, osteoradionecrosis of the jaw or odontogenic osteomyelitis of the jaw in our dental and oral surgery department between September 2021 and December 2022 were enrolled in this study. This study followed an observational, cross-sectional, within-subjects design. The permutation block randomization method with a block size of 4 was used to assign patients to the two groups.

### Inclusion criteria

The inclusion criteria were as follows: patients who (1) were diagnosed with osteomyelitis or osteonecrosis of the jaw; (2) who underwent bone SPECT to image the craniofacial region; (3) provided informed consent; (4) who were older than 20 years of age; (5) who consented to undergo bone scans twice on the same day with scan acquisition start times of 1.0 and 2.0-hour or 1.5-hour and 2.0-hour after injection of the radiopharmaceutical substance.

### Exclusion criteria

The exclusion criteria prior to data analysis were (1) high accumulation suggestive of parietal bone metastases on SPECT images, as the right and left parietal bones were treated as representative of healthy bones, or (2) no significant accumulation in the jaw lesions, or (3) presence of large metal implant within the jawbone lesions.

## MRONJ classification

Consistent with our previous study [11], MRONJ was classified according to the MRONJ staging system [13]. The diagnoses of MRONJ and cured MRONJ were performed by oral surgeons with more than 10 years of clinical experience.

###  Ethics approval consent to participate

This cross-sectional study was conducted in accordance with the Declaration of Helsinki, and the protocol was approved by the Hokkaido Cancer Center Hospital Ethics Review Board (registration numbers: 03–40). Although the total time commitment required for the two scans was within two hours and did not exceed the conventional duration, the possibility of an extended period of physical immobilization was anticipated. This was explained to the participants, and verbal consent for this was obtained with the approved of the Ethics Review Board. Verbal informed consent was obtained from all participants and was documented in their medical records.

### Data acquisition

Bone SPECT imaging was performed after an intravenous injection of 555 MBq [^99m^Tc] Tc-HMDP (Clearbone^®^ [^99m^Tc] Tc-HMDP; Nihon Medi-Physics Co., Ltd., Tokyo, Japan) via a SPECT dual-head gamma camera system (Discovery NM630; GE Healthcare, Chicago, IL, USA). The SPECT images were acquired in accordance with our previous study conditions and parameters [11]. The interval between the injection of the [^99m^Tc] Tc-HMDP and the start of acquisition of the first SPECT image was 1.0–1.5 h, and that for the second image was 2.0 h.

### Image reconstruction

The SPECT device used in this study was equipped with two detectors, each performing a 180° acquisition. The imaging protocol consisted of 30 steps with a view angle of 6° per step and a duration of 30 s per step. SPECT images were reconstructed using the ordered subset expectation maximization method and were smoothed using a three-dimensional spatial Gaussian filter.

### Data analysis

Data were analyzed using GI-BONE, a bone SPECT quantitative analysis software included in the medical device software package AZE Virtual Place Hayabusa (ver. 9.0; Nihon Medi-Physics Co., Ltd.). The first (i.e., 1.0-h or 1.5-h) image and the second 2.0-h image alignments were automatically co-regulated by the software. The SUVmax of the bilateral parietal bones were calculated by setting the fixed volume of interest (VOI) within a 12-pixel cube (62.1 cm3) [2, 11, 14]. Lesion site of the jawbone, the optimum threshold for the VOI was determined to be mean SUVmax of the right and left parietal bones added by 3 (+ 3). Based on this threshold, the VOI was semi-automatically calculated as metabolic bone volume (MBV) [2, 11, 14].

### Statistical analysis

Statistical analyses were performed via JMP^®^ Pro 16.0 software (SAS Institute Inc., Cary, NC, USA). The Wilcoxon rank-sum test was used to compare the SUVmax of the 1.0-hour image, 1.5-hour image, and 2.0-hour image. Statistical significance was set at *p* < 0.05. If significant differences were identified, the hypothesis was rejected. Conversely, if no statistically significant differences were observed, Bland‒Altman analysis was employed to assess equivalence.

## Results

### Patient characteristics

No patients were excluded from the 40 that were enrolled in the study. The 1.0-hour and 2.0-hour uptake groups (1.0-hour group: Patient 1to patient 20) included five men and 15 women, with a median (quartile 1, quartile 3) age of 64.5 (57.5, 72.3). The data for individual patients are presented in Table 1.

The 1.5-hour and 2.0-hour uptake groups (1.5-hour group: Patient 21 to patient 40) included seven men and 13 women with a median (quartile 1, quartile 3) age of 70.0 (62.3, 76.3). The data for individual patients are presented in Table 2.

The 1.0-hour group included 18 patients with MRONJ (10 with breast cancer, 3 with lung cancer, 3 with multiple myeloma, and 2 with prostate cancer), 1 patient with radiogenic osteonecrosis of the jaw, and 1 patient with odontogenic osteomyelitis. The 1.5-hour group included 18 patients with MRONJ (10 with breast cancer, 3 with lung cancer, 1 with multiple myeloma, 3 with prostate cancer, and 1 with osteoporosis) and two patients with odontogenic osteomyelitis.

### Comparison of the SUVmax within each group

The differences in the SUVmax between the 1.0-hour uptake time and 2.0-hour uptake time or between the SUVmax between the 1.5-hour uptake time and 2.0-hour uptake time were calculated at two sites (i.e., each patient’s parietal bones and jawbone lesions).

**Fig. 1:**
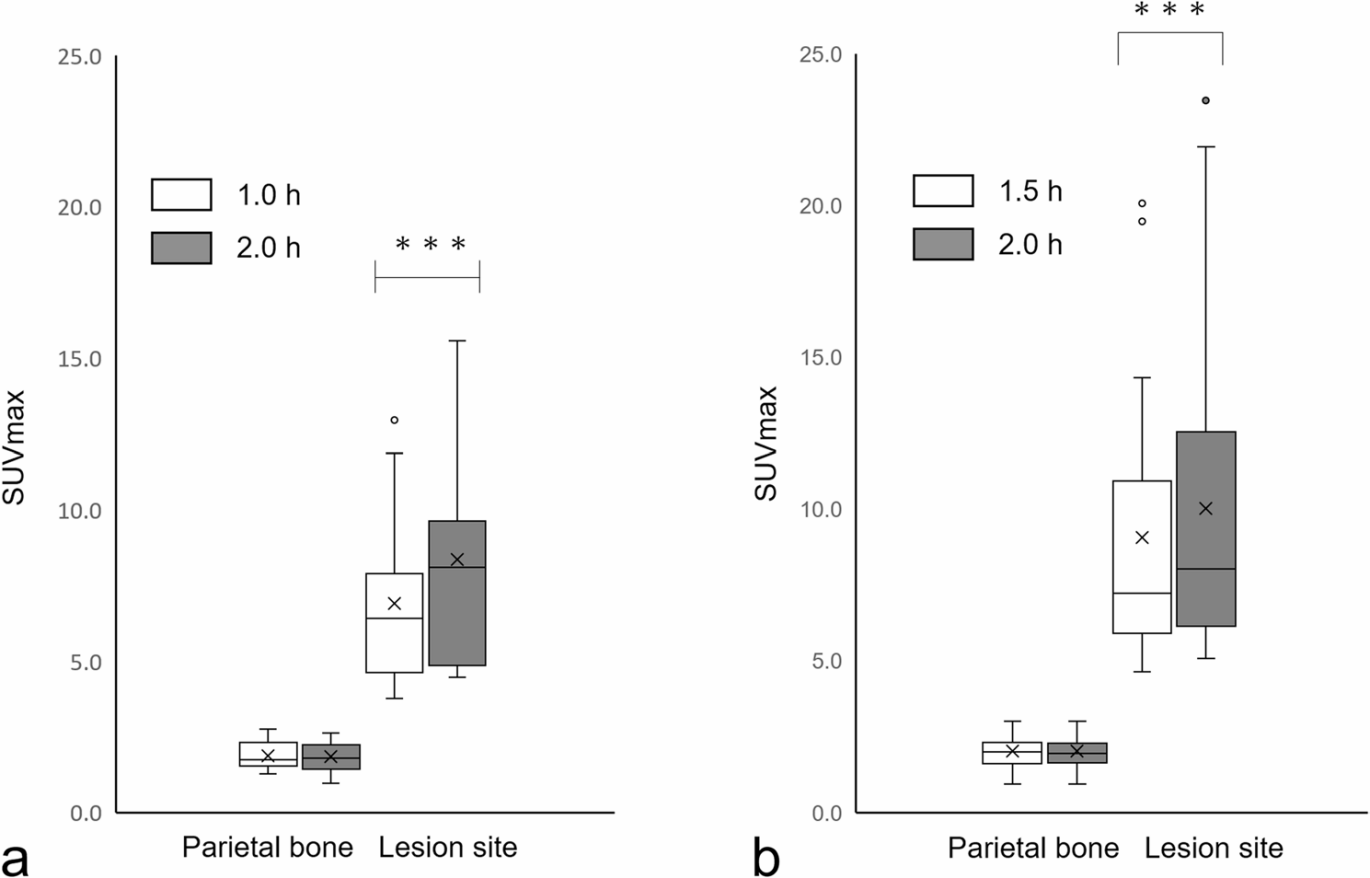
SUVmax for varying parietal bone and jawbone lesion SPECT imaging in the two groups. The median SUVmax (quartiles 1 and 3) of the parietal bones was 1.74 (1.55, 2.23), and it was 1.79 (1.42, 2.24) for the 1.0-h and 2.0-h images, respectively, with a median difference of −0.08. The median SUVmax of the jawbone lesions was 6.40 (4.61, 7.41) and 8.10 (5.14, 9.29) for the 1.0-h and 2.0-h images, respectively, with a median difference of 1.55 and a statistically significant difference (*p* < 0.001) (**a**). The median SUVmax (quartile 1, quartile 3) of the parietal bones was 1.98 (1.68, 2.30) and 1.93 (1.68, 2.22) for the 1.5-h and 2.0-h images, respectively, with a median difference of 0.04. The median SUVmax of the jawbone lesions was 7.22 (5.90, 10.84) and 8.03 (6.18, 12.38) for the 1.5-h and 2.0-h images, respectively, with a median difference of 0.94 and a statistically significant difference (*p* < 0.001) (**b**). Three asterisks (***) indicate a *p*-value of less than 0.001. *SUVmax*, maximum standardized uptake value; *SPECT*, single-photon emission computed tomography

A comparison of the SUVmax of the 1.0-hour group revealed that the median SUVmax values of the parietal bone were 1.74 and 1.79 for the 1.0-hour and 2.0-hour uptake values, respectively. No significant differences were observed between the two values (*p* = 0.121). The median SUVmax of jawbone lesions increased to 6.40 and 8.10, respectively, with a statistically significant difference (*p* < 0.001) (Fig. 1a). Similarly, in the 1.5-hour group, the median SUVmax of the parietal bone was 1.98 and 1.93 at 1.5 and 2.0 h, respectively, with no significant difference observed (*p* = 0.298). The median SUVmax of the jawbone lesions increased to 7.22 and 8.03, respectively, with a statistically significant difference (*p* < 0.001) (Fig. 1b). Due to the statistically significant differences between the two groups, equivalence comparisons were not conducted.

### The representative patients

Quantitative SPECT results of Patient 9, a representative patient in the 1.0-hour group, and Patient 21, a representative patient in the 1.5-hour group, are presented.

**Fig. 2:**
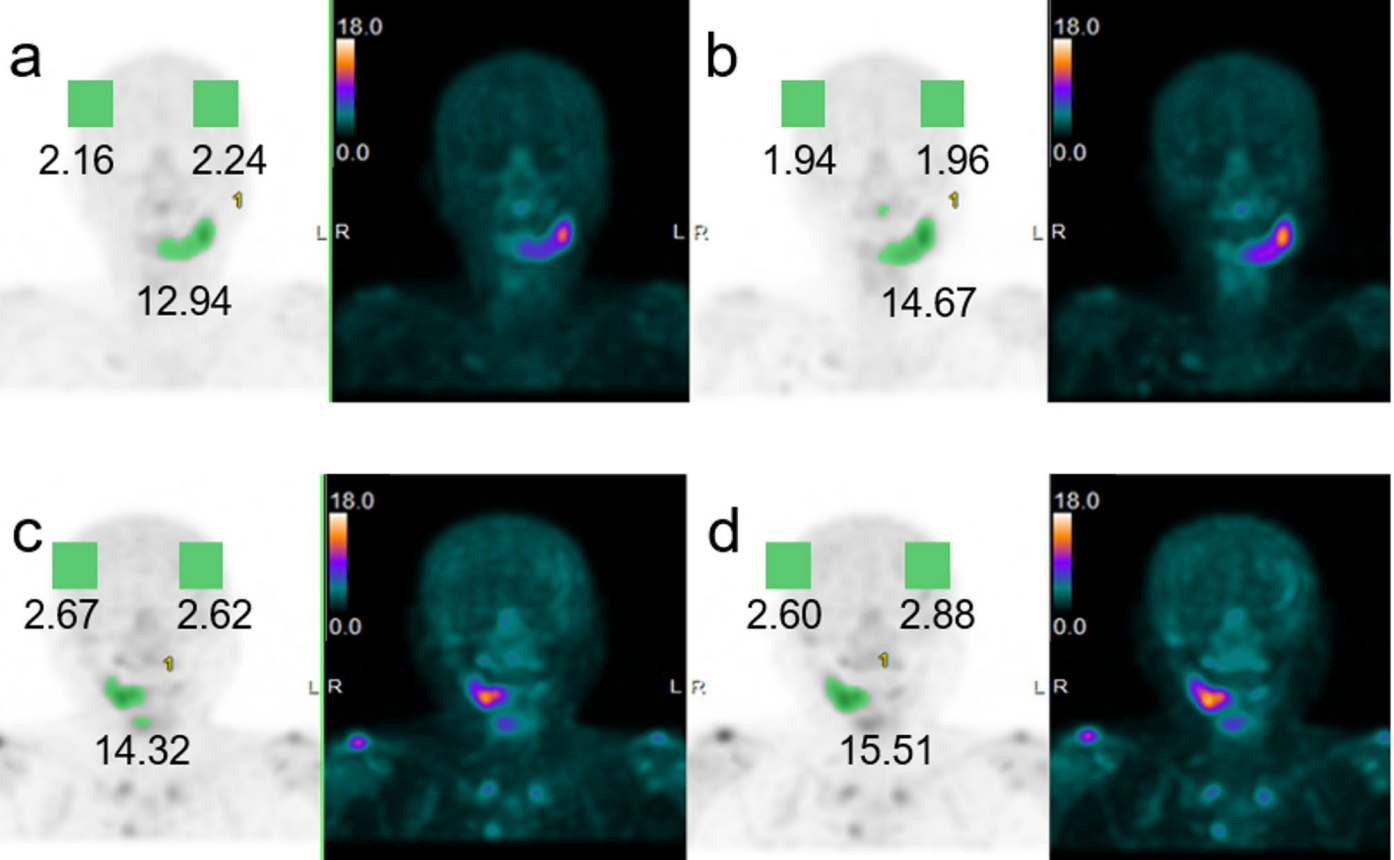
Depiction of representative cases (patient 9 and patient 21). Patient 9, representing the 1-hour uptake time group, was a 71-year-old woman. She received denosumab 20 times for bone metastases from breast cancer. She first presented to our department with the chief complaint of mandibular pain. She was diagnosed with stage 0 MRONJ with no obvious bone involvement in the left mandible. One-hour imaging revealed strong accumulation in the left mandible, with an SUVmax of 12.94 (**a**) that increased to 14.67 at 2 h (**b**). The average scores for the left and right parietal bones decreased slightly to −0.25. Patient 21, a 73-year-old woman, was representative of the 1.5-hour uptake time group. Swelling and drainage of pus from the fistula in the right mandibular gingiva with evidence of exposed necrotic bone. The patient was diagnosed with stage 2 MRONJ. In the 1.5-hour image, strong accumulation in the right mandible with an SUVmax of 14.32 was observed (**c**), and this increased to 15.51 in the 2.0-hour image (**d**). The average values for the left and right parietal bones increased slightly to 0.10. *SUVmax*, maximum standardized uptake value; *SPECT*, single-photon emission computed tomography

Patient 9 experienced Stage 2 MRONJ in the left mandible. The SUVmax of the left mandible was 12.94 at the 1-hour uptake time (Fig. 2a) but increased to 14.67 at the 2-hour uptake time (Fig. 2b). The average values of the left and right parietal bones decreased slightly, by −0.25 (Table 1). Patient 21 experienced stage 2 MRONJ in the right mandible. The SUVmax of the right mandible was 14.32 at 1.5 h (Fig. 2c) but increased to 15.51 at 2 h (Fig. 2 d). The average value of the left and right parietal bones increased slightly to 0.10 (Table 2).

**Table 1:** Characteristics of the patients in the 1.0-hour uptake time group and the SUVmax of the parietal bones and jawbone lesions

No.	Sex	Age (years)	Jawbone lesions (Cancer)	ARA	MRONJ stage	Jawbone	SUVmax of the parietal bones			SUVmax of the jawbone lesion		
							1.0 h	2.0 h	2.0 h–1.0 h	1.0 h	2.0 h	2.0 h–1.0 h
1	F	72	MRONJ (BC)	Dmab	2	Mandible	2.34	2.35	0.01	6.64	9.13	2.49
2	M	80	MRONJ (PC)	ZA	2	Mandible	1.27	0.96	−0.31	4.28	4.47	0.19
3	F	70	MRONJ (BC)	Dmab	3	Mandible	1.95	1.76	−0.19	6.32	7.76	1.44
4	F	60	MRONJ (BC)	ZA-Dmab	2	Mandible	1.39	1.26	−0.14	3.76	4.65	0.89
5	M	62	MRONJ (PC)	Dmab	2	Mandible	1.74	1.67	−0.07	11.86	15.57	3.71
6	F	67	MRONJ (LC)	Dmab	2	Mandible	1.75	1.82	0.06	6.54	7.58	1.04
7	F	73	MRONJ (BC)	Dmab	2	Mandible	2.49	2.63	0.14	8.13	9.78	1.65
8	M	58	OOJ	-	-	Mandible	2.36	2.18	−0.19	7.17	8.86	1.69
9	F	71	MRONJ (BC)	Dmab	1	Mandible	2.20	1.95	−0.25	12.94	14.67	1.73
10	F	93	MRONJ (BC)	Dmab	2	Maxilla	1.51	1.49	−0.03	11.49	13.40	1.91
11	F	49	ORNJ	-	-	Mandible	1.57	1.40	−0.17	6.88	8.70	1.82
12	M	58	MRONJ (LC)	Dmab	2	Mandible	2.51	2.31	−0.20	6.00	8.64	2.64
13	F	48	MRONJ (BC)	Dmab	Healed	Mandible	1.31	1.22	−0.09	4.33	4.58	0.25
14	F	55	MRONJ (BC)	Dmab	0	Mandible	1.62	1.61	−0.01	4.65	5.28	0.63
15	F	82	MRONJ (MM)	Dmab	1	Mandible	2.74	2.56	−0.18	5.80	6.24	0.44
16	F	76	MRONJ (LC)	Dmab	0	Mandible	1.56	1.73	0.17	6.35	7.37	1.02
17	F	61	MRONJ (MM)	ZA-Dmab	0	Mandible	1.95	1.96	0.01	4.62	4.72	0.10
18	M	56	MRONJ (MM)	Dmab	1	Mandible	2.08	2.24	0.16	6.45	8.44	1.99
19	F	55	MRONJ (BC)	Dmab	3	Maxilla	1.39	1.29	−0.10	4.14	4.50	0.36
20	F	72	MRONJ (BC)	ZA	3	Maxilla	1.70	2.24	0.54	9.81	12.52	2.71
median		64.5			2		1.74	1.79	−0.08	6.40	8.10	1.55
Q1-Q3		(57.5–72.3)					(1.55–2.23)	(1.42–2.24)	(−0.18-0.02)	(4.61–7.41)	(5.14–9.29)	(0.58–1.93)

*ARA* antiresorptive agent, *Dmab* Denosumab, *SUVmax* maximum standardized uptake value, *MRONJ* medication-related osteonecrosis of the jaw, *OOJ* odontogenic osteomyelitis of the jaw, *ORNJ* osteoradionecrosis of the jaw, *Q1* quartile 1, *Q3* quartile 3, *M* male, *F* female, *ZA* Zoledronic acid, *BC* Breast cancer, *LC* Lung cancer, *MM* Multiple myeloma, *PC* Prostate cancer

**Table 2:** Characteristics of the patients in the 1.5-hour uptake time group and the SUVmax of the parietal bones and jawbone lesions

No.	Sex	Age	Jawbone lesions (Cancer)	ARA	MRONJ stage	Jawbone	SUVmax of the parietal bones			SUVmax of the jawbone lesion		
							1.5 h	2.0 h	2.0 h–1.5 h	1.5 h	2.0 h	2.0 h–1.5 h
21	F	73	MRONJ (BC)	Dmab	2	Mandible	2.65	2.74	0.10	14.32	15.51	1.19
22	F	57	MRONJ (BC)	ZA	1	Mandible	2.17	1.94	−0.23	6.47	6.20	−0.27
23	F	70	MRONJ (BC)	Dmab	2	Mandible	1.77	1.85	0.09	5.90	6.12	0.22
24	M	73	MRONJ (MM)	Dmab	2	Mandible	1.73	1.72	−0.01	13.47	15.50	2.03
25	F	66	MRONJ (BC)	ZA	2	Maxilla	2.11	2.20	0.09	6.61	7.64	1.03
26	F	70	MRONJ (BC)	Dmab	2	Maxilla	3.00	3.00	0.00	8.23	8.16	−0.07
27	M	80	MRONJ (LC)	Dmab	0	Mandible	1.55	1.59	0.04	7.58	8.44	0.86
28	F	70	MRONJ (BC)	ZA	2	Mandible	2.68	2.73	0.06	6.86	7.90	1.04
29	M	80	MRONJ (PC)	ZA	Healed	Mandible	0.94	0.93	−0.01	4.88	5.06	0.18
30	F	60	MRONJ (BC)	ZA-Dmab	2	Mandible	1.40	1.59	0.19	5.46	5.20	−0.26
31	F	67	MRONJ (LC)	Dmab	2	Mandible	1.84	1.93	0.09	4.62	5.87	1.25
32	M	75	MRONJ (PC)	Dmab	3	Mandible	1.41	1.47	0.06	8.33	9.34	1.01
33	M	81	MRONJ (LC)	Dmab	2	Mandible	1.79	1.77	−0.02	10.80	12.58	1.78
34	F	49	OOJ	-	-	Maxilla	2.17	2.10	−0.07	5.86	6.61	0.75
35	M	63	MRONJ (PC)	Dmab	2	Mandible	1.54	1.59	0.05	10.97	12.31	1.34
36	M	58	OOJ	-	-	Mandible	2.29	2.17	−0.12	6.08	6.55	0.47
37	F	90	MRONJ (BC)	Dmab	3	Mandible	2.88	2.92	0.04	20.05	21.91	1.86
38	F	71	MRONJ (BC)	Dmab	2	Mandible	2.32	2.18	−0.14	19.46	23.44	3.98
39	F	57	MRONJ (BC)	ZA-Dmab	3	Mandible	1.85	1.87	0.02	5.89	6.08	0.19
40	F	95	MRONJ Osteoporosis	IA	2	Mandible	2.18	2.29	0.11	9.50	9.93	0.43
median		70			2		1.98	1.93	0.04	7.22	8.03	0.94
Q1-Q3		(62.3–76.3)					(1.68–2.30)	(1.68–2.22)	(−0.01-0.09)	(5.90-10.84)	(6.18–12.38)	(0.21–1.27)

*ARA* antiresorptive agent, *Dmab* denosumab, *IA* ibandronic acid, *SUVmax* maximum standardized uptake value, *MRONJ* medication-related osteonecrosis of the jaw, *OOJ* odontogenic osteomyelitis of the jaw, *Q1* quartile 1, *Q3* quartile 3, *M* male, *F* female, *ZA* Zoledronic acid, *BC* Breast cancer, *LC* Lung cancer, *MM* Multiple myeloma, *PC* Prostate cancer

## Discussion

To the best of our knowledge, no prior study other than our previous study [11] has evaluated the optimal uptake time for bone SPECT via a quantitative evaluation of the craniomaxillofacial bones. In that study, we reported that the SUVmax of 2-hour and 3-hour images of the parietal bone and jawbone lesions via quantitative SPECT using a [^99m^Tc] Tc-HMDP was clinically equivalent and that the uptake time could be shortened to 2 h. In this study, we investigated whether it is possible to further reduce the uptake time. The average uptake time for imaging of planar images performed at 25 nuclear medicine centers in a Swedish multicenter study was 3.04 [min = 2, max = 4] hours [15]. Although there are variations among facilities, it is likely that the EANM guidelines of 2–5 h from injection to image acquisition [9] are often adhered to. Accurate selection of the optimal uptake duration is essential for obtaining optimal image quality and can affect diagnoses and recommended courses of treatment. Tc-99 m-labeled diphosphonate allows clearance of the radiotracer from soft tissues during the uptake time, thus increasing the target-to-background ratio and improving bone visualization [16, 17]. The background was not emphasized in either the 1.0-hour group or the 1.5-hour group compared to the 2.0-hour uptake time image.

After intravenous administration of diphosphonates, about 50–60% binds to the skeleton within 4 h, 34% is excreted in urine, and 6% remains in circulation. Bone accumulation peaks 1-hour post-injection and stays constant for up to 72 h [9].

Although our two groups targeted different patients, the median (IQR) SUVmax of the jawbone lesion at 2 h was 8.10 [5.14, 9.29] and 8.03 [6.18, 12.3], respectively, and there was no significant difference between the groups, indicating that both groups consisted of populations with jawbone lesions exhibiting a similar degree of inflammatory activity. When the 1.0-hour group and the 1.5-hour group were compared, the median difference between the SUVmax of the jawbone lesion and the 2-hour value decreased from 1.55 to 0.94. This finding suggests that the accumulation of [^99m^Tc] Tc-HMDP in the lesion was still ongoing and had not yet reached a plateau at 1–1.5 h. The data obtained in the present study corroborate the EANM recommendations for shortest uptake time in bone scintigraphy.

### Limitations

Our study possesses several limitations. First, it included a relatively small number of enrolled patients and utilized a single-center cross-sectional study design. A multicenter study with a larger sample size could provide additional information regarding different imaging protocols. Second, a stand-alone SPECT device was used in this study. Therefore, CT-based attenuation correction or scatter correction was not performed, and this may have resulted in inferior SUV accuracy. In addition, the exclusive use of SUVmax, a parameter that is highly susceptible to image noise, may have further compromised the precision of the quantitative assessment. Third, in this study, the radioactivity of the syringe after tracer injection was not measured; therefore, the exact injected dose remains unknown. This limitation may have affected the accuracy of the SUV assessment. Fourth, due to the nature of this study as a clinical investigation involving human subjects, it was not feasible to acquire data at temporal intervals shorter than 30 min. Additional research is warranted to determine whether the 2-hour uptake time can be further reduced.

### Potential for generalizing the results

First, our study was limited to quantitative SPECT findings for parietal bone and jawbone lesions (each of which is relatively close to the body surface). Thus, additional validation is needed to determine whether the present results can be generalized and adapted for other bone sites. Second, the bone tracer used in this study was [^99m^Tc] Tc-HMDP that is known to exhibit a rapid bone uptake time. Additional verification is warranted to confirm whether similar results can be obtained for other types of MDP and other bone tracers.

## Conclusions

We determined that the limit for shortening the uptake time was 2 h when follow-up was performed with a stable SUVmax on quantitative SPECT of the jawbone.

## Data Availability

The authors have full control of all primary data and have agreed to allow the journal to review the data if requested. The datasets used and/or analyzed during the current study are available from the corresponding author on reasonable request.

## Abbreviations

ARA: antiresorptive agent
BCF: Becquerel calibration factor
HMDP: hydroxymethylene diphosphonate
LOA: limit of agreement
MDP: methylene diphosphonate
MRONJ: medication-related osteonecrosis of the jaw
SPECT: single-photon emission computed tomography
SUVmax: maximum standardized uptake value
Tc-99 m: technetium-99 m
